# Moderate physical activity and higher frequency are inversely associated with incidence of frailty in middle-aged and older population: a 4-year longitudinal study in Europe

**DOI:** 10.1007/s41999-024-01073-z

**Published:** 2024-10-01

**Authors:** Fanji Qiu, Yichao Yu, Jinfeng Li

**Affiliations:** 1 Movement Biomechanics, Institute of Sport Sciences, Humboldt-Universität zu Berlin, Unter den Linden 6, 10099 Berlin, Germany; 2 School of Sports Coaching, Beijing Sports University, Beijing, 100084 China; 3 Department of Kinesiology, Iowa State University, Ames, IA 50011 USA

**Keywords:** Exercise, Frailty, Middle-aged, Older, Incidence

## Abstract

**Background:**

Global aging is leading to an increase in frailty patients, and physical activity (PA) may have an impact on frail in middle-aged and older population. This study aimed to explore the relationship between the frequency of different PA intensities and the incidence and prevalence of frailty in middle-aged and older adults based on the Survey of Health, Ageing, and Retirement in Europe (SHARE).

**Method:**

Self-reported questionnaires were used to obtain information on demographics and PA, and frailty was assessed using the SHARE Frailty Instrument (SHARE-FI). Cox regression and logistic regression models were used to explore the association between PA and frailty and stratified according to middle or old age.

**Results:**

Among 6315 baseline non-frail participants aged 44–96 years, 16.1% developed frailty over 4 years, with higher incidence and prevalence in women (*P* < 0.05). Women accounting for 55.80% of the sample. The frequency of participants participating in sports decreased over 4 years (*P* < 0.05). Compared with participants who engaged in PA more than once a week, participants who engaged in moderated PA less frequently had a higher risk of new-onset frailty (HR: 3.174–6.115), and participants who engaged in vigorous PA 1–3 times a month had a higher risk of new-onset frailty (HR: 1.335). Participation in low-frequency moderate PA and vigorous PA 1–3 times per month were positively associated with the prevalence of frailty (*P* < 0.05).

**Conclusion:**

Physical activity frequency decreases with age in middle-aged and older adults. Those adults who engage in moderate PA more than once a week have a lower risk of incidence of frailty, compared to those with more sedentary life-styles. Additionally, women need to pay more attention to frailty management.

## Key summary points

**Aim:**

In the context of global aging, further exploration is needed to understand the associations of varying exercise intensities and frequencies on frailty among middle-aged and older individuals.

**Findings:**

As people age, their participation in physical activity tends to decrease. A longitudinal association was observed between moderate-intensity physical activity more than once a week and lower incidence of frailty among middle-aged and older adults, compared to participants with lower frequency of moderate physical activity.

**Message:**

Further interventional studies are needed to clarify the causal relationship between different intensities of physical activity and preventing frailty.

## Introduction

Frailty is defined as a state in which the function and reserve capacity of multiple physiological systems of the body are diminished, making the body more vulnerable to stressors [1]. It will increase the risk of disability, chronic disease morbidity, and mortality. Common characteristics of frailty include muscle weakness, cognitive impairment, susceptibility to fatigue, reduced exercise capacity, unintentional weight loss, and even reduced ability of the body to recover from illness[2, 3]. Among individuals aged 60 and above, the incidence of frailty is 13.6% [4], with prevalence rates ranging from 7.7% to 39.2% [5, 6]. The prevalence of frailty increases with age [7], suggesting that an increasing number of middle-aged and older adults will encounter frailty-related challenges in the future. Therefore, enhancing focus on frailty within this demographic is crucial for better prevention and management, ultimately improving their quality of life.

The state of frailty is reversible, and its progression can be slowed or delayed through interventions [8, 9]. Physical activity (PA) is one of the effective potential strategies for preventing or reversing frailty. PA can positively impact immunity, skeletal muscle function, and neuromuscular control by improving vascular function and antioxidant capacity [9, 10].

Exercise plays a crucial role in both preventing and reversing frailty among community-dwelling adults (8)[8]. For individuals aged 18–65, engaging in at least 150 min of moderate-intensity exercise weekly is beneficial for maintaining good health [11]. There is a dose–response relationship between the volume of exercise and its health benefits; increased exercise volume enhances muscle capacity [12]. A randomized-controlled trial (RCT) involving 163 frail adults aged 65 and older demonstrated the positive effects of exercise-based interventions on frailty. After participating in a 24-week mixed exercise program, 41.7% of the initially frail participants had reversed their frailty status [13]. The benefits of sustained physical activity were also evident in the long term. In a 1-year follow-up study with 1,735 participants with an average age of 79.6, those who reduced their frequency of moderate physical activity exhibited more severe signs of frailty—physically, psychologically, and socially—compared to those who maintained regular activity [14]. In cohort studies, the association between exercise and frailty has also been observed. An inverse association between engaging in at least 150 min of PA per week and the severity of frailty was found in a cross-sectional study involving 3,758 participants aged 60 and above [15]. In a longitudinal cohort study, which included 7,006 participants who were non-frail at baseline with a mean age of 80.6, observed that sustained aerobic exercise reduced frailty risk by 26% over a 3.1-year follow-up [16]. Additionally, a cohort study of 601 older participants with a mean age of 84 found an inverse association between self-reported leisure-time physical activity and frailty after 12–14 years of follow-up [17]. However, these studies only focused on whether or not people participated in exercise, or on a single intensity of exercise. Thus, further research is necessary to explore the association between frequency and intensity of exercise and frailty incidence in middle-aged and older cohorts.

This study aims to examine the association between physical activity frequency of different intensities and new onset of frailty. We hypothesize that: 1. the likelihood of developing new-onset frailty increases as PA frequency decreases among middle-aged and older adults, and 2. higher levels of PA are associated with lower incidence and prevalence of frailty.

## Method

### Study population

**Fig. 1:**
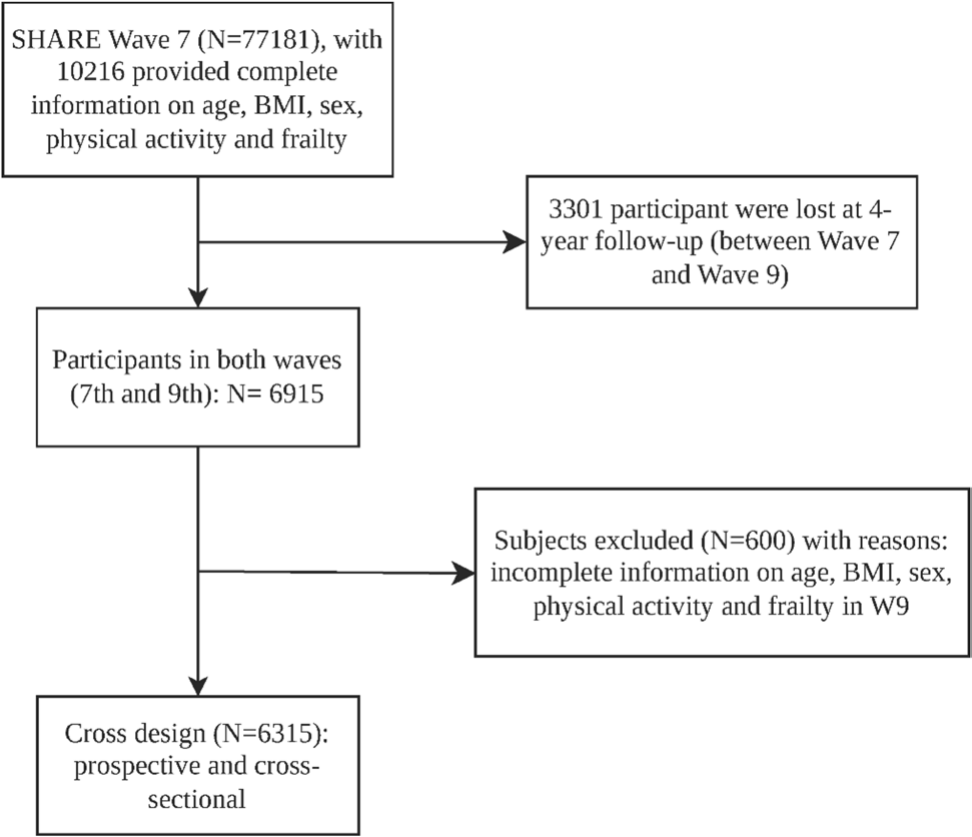
Flow diagram of data selection

This prospective cohort study utilized data from the 7th and 9th waves of the Survey of Health, Ageing and Retirement in Europe (SHARE). SHARE is a longitudinal population study database that, between the seventh and ninth waves, included data from over 80,000 adults aged 50 and above, as well as some of their family members, across 27 European countries and Israel. The database captures multidisciplinary data on health and socioeconomic status through computer-assisted personal interviewing (CAPI) and questionnaires [18–20]. Due to the interruption caused by the global COVID-19 pandemic, the data from the 8th wave were unavailable, leading to the use of data from the 7th (beginning in 2017) and 9th (beginning in 2021) waves. Across these waves, approximately 140,000 interviews were conducted, with a final dataset including 6,315 participants (Fig. 1). All participants provided information on frailty status, physical activity participation, and demographics. At the outset (wave 7), participants were classified as “non-frail”, “pre-frail”, and “frail”. We combined the “non-frail” and “pre-frail” categories into “non-frail” for statistics. To minimize bias from missing data, participants with incomplete data were excluded. The SHARE study obtained ethical approval from the ethics committees of the participating countries, and all participants provided written informed consent [19].

### Measurement of frailty

Frailty status in participants was assessed at baseline (wave 7) and wave 9 using the SHARE Frailty Instrument (SHARE-FI) [21]. The SHARE-FI, developed based on the SHARE database, evaluates frailty through five components: “EXHAUSTION,” “LOSS OF APPETITE,” “WEAKNESS,” “WALKING DIFFICULTIES,” and “LOW PHYSICAL ACTIVITY.” “WEAKNESS” was assessed by measuring the participant’s maximum grip strength, while the other components are evaluated using data collected from the SHARE questionnaire. The results from the SHARE-FI categorize participants into three groups: “NON-FRAIL,” “PRE-FRAIL,” and “FRAIL”.

### Physical activity

Information on physical activity (PA) was obtained through a self-reported questionnaire. Participants in the SHARE survey were invited to answer the following two questions to assess the intensity and frequency of their daily exercise: 1. “How often do you engage in vigorous physical activity, such as sports, heavy housework, or a job that involves physical labor?” 2, “How often do you engage in activities that require a moderate level of energy such as gardening, cleaning the car, or taking a walk?”. The frequency of PA was categorized into four levels: 1. More than once a week, 2. Once a week, 3. One to three times a month, and 4. Hardly ever or never.

### Covariates

Covariates included participants’ demographic information collected through questionnaires, including age, body mass index (BMI), and sex.

### Statistical analysis

Data processing was conducted using SPSS (version 26, IBM: Armonk, NY, USA). Sample size calculations were based on Cochran’s sample size formula, assuming a frailty incidence rate of 13.6% [4], and with a 95% confidence interval and a β error of 5%. The calculated minimum sample size was 181 subjects [22]. Multicollinearity analysis was assessed among the variables, with strong multicollinearity defined as a maximum variance inflation factor (VIF) value greater than 5 and a tolerance value less than 0.20 [23, 24]. Baseline characteristics of the study population, including age (middle-aged: 40–65 years, older: > 65 years), BMI, and sex, were described using descriptive analysis. Continuous variables were presented as means with standard deviations (SD). Group differences were evaluated using independent sample t tests or Mann–Whitney U test for continuous variables and χ2 tests for categorical variables. Cox regression analyzed the association between different PA frequencies and frailty, while binary logistic regression assessed the cross-sectional association between PA frequency and frailty. Hazard ratios (HR), odds ratios (OR), 95% confidence intervals, and *P* values (*P* < 0.05 indicating statistically significant) were calculated. Models were stratified by PA frequency, with the reference categories being engagement in vigorous/moderate PA more than once a week and male sex.

## Results

### Descriptive participant characteristics

A total of 6,315 participants, aged 44–96 years, were included in this study with women accounting for 55.80% of the sample. The follow-up period for participants was 4 years, during which the incidence of new-onset frailty was 16.09% (Table 1). Cox regression revealed no significant difference in the incidence of frailty between middle-aged (15.05%) and older adults (16.49%). Participants with new-onset frailty were older, had a higher BMI, and included a higher proportion of women compared to those without frailty. Additionally, there was a notable decline in the participation of both vigorous and moderate PA among participants over the 4-year period. Collinearity diagnostics indicated no multicollinearity among variables (VIF < 5, Tolerance > 0.1).

**Table 1:** The characteristics of participants at wave 9

Variables	All			Middle-aged			Older		
	All	Frailty	Non-frailty	All	Frailty	Non-frailty	All	Frailty	Non-frailty
Age	6315	1016	5299	1767	266	1501	4548	750	3798
	70.24 (7.05)	70.76 (7.51)	70.15 (6.96)*	62.27 (2.77)	62.23 (2.58)	62.27 (2.80)	73.34 (5.63)	73.78 (6.25)	73.26 (5.50)*
BMI	26.79 (4.23)	27.05 (4.29)	26.74 (4.22)*	26.79 (4.56)	27.30 (4.62)	26.70 (4.55)	26.80 (4.10)	26.97 (4.17)	26.76 (4.08)
Sex (female)	3524 (55.80)	790 (77.76)	2734 (51.59)*	1149 (65.03)	198 (74.44)	951 (63.36)*	2375 (52.22)	592 (78.93)	1783 (46.95)*
Vigorous									
More than once a week	1675 (26.52)	141 (13.88)	1534 (28.95)*	591 (33.45)	53 (19.92)	538 (35.84)*	1084 (23.83)	88 (11.73)	996 (26.22)*
Once a week	839 (13.29)	105 (10.33)	734 (13.85)*	288 (16.30)	25 (9.40)	263 (17.52)*	551 (12.12)	80 (10.67)	471 (12.40)
1–3 times a month	809 (12.81)	145 (14.27)	664 (12.53)	260 (14.71)	46 (17.29)	214 (14.26)	549 (12.07)	99 (13.20)	450 (11.85)
Hardly ever, or never	2992 (47.38)	625 (61.52)	2367 (44.67)*	628 (35.54)	142 (53.38)	486 (32.38)*	2364 (51.98)	483 (64.40)	1881 (49.53)*
Moderate									
More than once a week	4435 (70.23)	338 (33.27)	4097 (77.32)*	1348 (76.29)	93 (34.96)	1255 (83.61)*	3087 (67.88)	245 (32.67)	2842 (74.83)*
Once a week	781 (12.37)	190 (18.70)	591 (11.15)*	201 (11.38)	48 (18.05)	153 (10.19)*	580 (12.75)	142 (18.93)	438 (11.53)*
1–3 times a month	372 (5.89)	161 (15.85)	211 (3.98)*	88 (4.98)	51 (19.17)	37 (2.47)*	284 (6.24)	110 (14.67)	174 (4.58)*
Hardly ever, or never	727 (11.51)	327 (32.19)	400 (7.55)*	130 (7.36)	74 (27.82)	56 (3.73)*	597 (13.13)	253 (33.73)	344 (9.06)*

*BMI* body mass index (Kg/m^2^)

*There was a significant difference between the two groups, *P* < 0.05. The categorical variable (sex) was analyzed using Chi-square test, while the continuous variables (age and BMI) were analyzed using Mann–Whitney *U* test

Age range (years): all participants: 44–96; middle-aged: 44–65; older: 66–96

### Results of Cox regression

The Cox regression analysis (Table 2) demonstrated an inverse dose–response relationship between the frequency of moderate PA and the risk of developing new-onset frailty among all participants. Specifically, women were at a significantly higher risk of developing frailty compared to males (HR = 2.779, *P* < 0.05). Participants who engaged in PA 1–3 times per month had a significantly higher risk of new-onset frailty compared to those who participated in PA more than once a week (HR = 1.335, *P* < 0.05). For moderate PA, participants who exercise less frequently are at increased risk for new frailty compared with participants who exercise more than once a week (*P* < 0.05). However, age and BMI did not show a significant association with new-onset frailty.

**Table 2:** Association between frequency of PA and frailty from Cox regression (hazard ratios)

Variables	All		Middle-aged		Older	
	HR (95%CI)	*P* value	HR (95%CI)	*P* value	HR (95%CI)	*P* value
Age	1.000 (0.991, 1.008)	0.910	1.013 (0.969, 1.058)	0.574	0.996 (0.983, 1.008)	0.511
BMI	1.001 (0.988, 1.015)	0.842	1.002 (0.978, 1.026)	0.887	1.000 (0.984, 1.017)	0.990
Sex*	2.779 (2.394, 3.227)	< 0.001	1.545 (1.164, 2.051)	< 0.01	3.395 (2.846, 4.049)	< 0.001
Vigorous						
More than once a week	1 (reference)		1 (reference)		1 (reference)	
Once a week	1.168 (0.906, 1.508)	0.231	0.790 (0.489, 1.276)	0.335	1.419 (1.045, 1.926)	0.025
1–3 times a month	1.335 (1.050, 1.696)	0.018	1.285 (0.856, 1.929)	0.227	1.366 (1.015, 1.839)	0.040
Hardly ever, or never	1.049 (0.855, 1.287)	0.648	1.021 (0.714, 1.460)	0.909	1.072 (0.835, 1.377)	0.586
Moderate						
More than once a week	1 (reference)		1 (reference)		1 (reference)	
Once a week	3.174 (2.646, 3.809)	< 0.001	3.490 (2.450, 4.972)	< 0.001	3.033 (2.453, 3.751)	< 0.001
1–3 times a month	5.421 (4.453, 6.600)	< 0.001	7.806 (5.441, 11.197)	< 0.001	4.674 (3.696, 5.912)	< 0.001
Hardly ever, or never	6.115 (5.131, 7.287)	< 0.001	8.059 (5.667, 11.460)	< 0.001	5.642 (4.609, 6.907)	< 0.001

*HR* Hazard ratios

Cox regression models were adjusted for co-variates: age (years), sex (Male, Female), BMI (Kg/m^2^)

*Male as reference

When stratified by age, both middle-aged and older populations exhibited a similar pattern, where lower frequencies of moderate PA were linked to a higher risk of developing new-onset frailty, compared to those engaging in PA more than once a week (*P* < 0.05). For the older population, engaging in vigorous PA 1–3 times per month was associated with a higher risk of new-onset frailty compared to more than once a week (*P* < 0.05).

### Results of logistic regression

According to the results of the binary logistic regression analysis (Table 3), an inverse dose–response relationship was observed between the frequency of moderate PA and frailty prevalence for all participants, and female sex was positively associated with frailty prevalence compared to engaging in PA more than once a week (*P* < 0.05). Additionally, engaging in vigorous PA 1–3 times per month was positively associated with higher frailty prevalence (*P* < 0.05). Similar to the Cox regression findings, age and BMI were not significantly associated to frailty prevalence.

**Table 3:** Association between frequency of PA and frailty from multivariate binary logistic regression (Odds ratios)

Variables	All		Middle-aged		Older	
	OR (95%CI)	*P* value	OR (95%CI)	*P* value	OR (95%CI)	*P* value
Age	0.999 (0.989, 1.010)	0.914	1.021 (0.967, 1.078)	0.457	0.993 (0.978, 1.009)	0.396
BMI	1.003 (0.990, 1.016)	0.627	1.005 (0.978, 1.032)	0.732	1.001 (0.985, 1.016)	0.945
Sex*	4.094 (3.440, 4.872)	< 0.001	1.907 (1.354, 2.685)	< 0.001	5.255 (4.287, 6.442)	< 0.001
Vigorous						
More than once a week	1 (reference)		1 (reference)		1 (reference)	
Once a week	1.195 (0.898, 1.589)	0.222	0.736 (0.432, 1.252)	0.258	1.542 (1.092, 2.176)	< 0.05
1–3 times a month	1.462 (1.111, 1.925)	< 0.01	1.421 (0.888, 2.275)	0.143	1.505 (1.070, 2.117)	< 0.05
Hardly ever, or never	1.030 (0.819, 1.296)	0.798	1.001 (0.663, 1.510)	0.996	1.056 (0.798, 1.397)	0.703
Moderate						
More than once a week	1 (reference)		1 (reference)		1 (reference)	
Once a week	4.168 (3.382, 5.137)	< 0.001	4.384 (2.948, 6.521)	< 0.001	4.080 (3.186, 5.225)	< 0.001
1–3 times a month	10.179 (7.891, 13.131)	< 0.001	17.832 (10.866, 29.263)	< 0.001	8.389 (6.202, 11.346)	< 0.001
Hardly ever, or never	12.397 (9.944, 15.454)	< 0.001	18.377 (11.565, 29.202)	< 0.001	11.511 (8.925, 14.845)	< 0.001

*OR* Odds ratio

Logistic regression models were adjusted for co-variates: age (years), sex (Male, Female), BMI (Kg/m^2^)

*Male as reference

When stratified by age, both middle-aged and older populations demonstrated that lower frequencies of moderate PA were positively associated with frailty prevalence compared to engaging in PA more than once a week (*P* < 0.05). Moreover, engaging in vigorous PA once a week or 1–3 times per month were associated with higher frailty prevalence (*P* < 0.05).

## Discussion

Our study reveals that among middle-aged and older adults, lower frequencies of moderate PA participation and women are associated with higher incidence and prevalence of frailty. Furthermore, across different age groups, the incidence and prevalence of frailty is consistently higher in women than in men.

It is worth noting that there is a causal inverse relationship between PA frequency and the incidence of new-onset frailty. The health benefits of exercise for middle-aged and older adults are well documented. Recommendations from the U.S. Department of Health and Human Services shown that engaging in balance training, resistance training, and moderate-intensity aerobic exercise for 30 to 45 min, at least three times a week, for a duration of at least 3 to 5 months could significantly enhance the functional capacity of frail older adults [25]. Furthermore, a meta-analysis on resistance training in frail older adults found that training 1–6 times per week, with training volumes of 1–3 sets, 6–15 repetitions per session at an exercise intensity of 30–70% one-repetition maximum, effectively improved muscle strength and functional outcomes [26]. Results from cohort studies have already demonstrated a cross-sectional inverse association between PA and frailty. A cross-sectional study of 638 participants with an average age of 77 years demonstrated that higher PA participation duration, measured by accelerometers, less sedentary time, and more frequent physical activity were significantly associated with lower frailty prevalence [27]. Another study involving 511 older adults, with an average age of 73.4 years, found that frail or pre-frail individuals spent less time in moderate-to-vigorous physical activity (MVPA) and higher sedentary time compared to their healthier peers [28]. A UK-based longitudinal study showed that engaging in moderate PA at least once a week reduces frailty among individuals aged 65 and older [29]. Our findings extend these observations to the middle-aged population, indicating that a lower frequency of moderate PA associates with higher frailty incidence in middle-aged and older adults. Therefore, more frequent participation in moderate-intensity PA is an important measure to reduce the new onset of frailty.

Middle-aged and older individuals should actively engage in moderate levels of physical activity. A decrease in the frequency of physical activity participation among middle-aged and older individuals as they age has been observed in our study, with frailty patients exhibiting significantly lower physical activity frequency compared to non-frailty adults. Similar changes have been observed in studies of healthy participants. A survey of over 90,000 individuals in England found that both the level of exercise participation and willingness to engage in exercise decreases with age [30]. Furthermore, individuals aged 70–79 are about half as likely to engage in high-level physical activity as those aged 50–59, and individuals over 80 are even less than half as likely to participate in sports or express a desire to increase their activity levels compared to those in their early 50 s [31]. Despite this decline, moderate-intensity exercise is generally well tolerated by middle-aged and older adults, with fewer adverse events reported. For example, an RCT involving 100 sedentary women aged 49–82 found that during a 12 months of moderate-intensity exercise program, fewer than 10% of participants reported adverse events, which were primarily minor issues like muscle soreness [32]. Furthermore, a large-scale retrospective study involving 6 million participants, with an average age of 43.8, indicated that the higher frequencies of moderate physical activity were associated with fewer cardiovascular-related adverse events [33]. Therefore, frailty patients should prioritize engaging in moderate-intensity physical activity.

Although the relationship between vigorous exercise and frailty has been less frequently studied, this does not imply that vigorous exercise has limited effects on improving frailty. An RCT involving 94 frail participants with an average age of 87.1 found that a 10-week high-intensity, progressive resistance training program significantly improved muscle strength and gait speed, largely due to increases in muscle cross-sectional area [34]. Another study involving 169 individuals aged 65 and older demonstrated that 12 weeks of high-intensity functional training (HIFT) improved functional capacity, balance, and gait speed in frail older individuals [35]. Consequently, vigorous-intensity exercise should be considered an important strategy for enhancing health outcomes in middle-aged and older frail individuals.

Women require more attention in terms of frailty. Gender differences in frailty have been observed in cross-sectional studies from SHARE, which show a positive association between female sex and frailty prevalence in populations aged 50 and above [36]. In a cohort study including 1,104 participants aged 65 and above for 3.04 years, it was also observed that women had a higher incidence of frailty than men regardless of the assessment tool used to determine frailty [37]. The mortality-incidence paradox [38] explains why the incidence of frailty is higher in women than men. This paradox suggests that although women have slightly poorer average health compared to men, they have a longevity advantage. This may be due to the higher mortality rate of men compared with women, resulting in the surviving males in better health as they aged [39]. Therefore, more efforts are needed to understand the frailty status of women to provide targeted prevention and treatment measures.

This cohort study with a 4-year follow-up period in middle-aged and older adults provided the opportunity to investigate the association between PA frequency and subsequent new-onset frailty. Evidence further supports the dose–response associations between chronic disease and new-onset frailty.

Our study presents several limitations. First, the observational design precludes the establishment of causality between physical activity and the incidence of frailty. Second, since participants were exclusively from European countries and Israel, and given the disparities in physical activity levels observed across these countries [40], our findings may not be applicable to populations outside these regions. Third, the increased mortality risk among frail participants may have led to attrition [41], potentially causing an underestimation of the associations between physical activity and frailty. Fourth, PA was categorized into only four levels, which does not allow for precise calculations of exercise dosage appropriate for middle-aged and older adults. Finally, the reported statistics on the incidence and prevalence of frailty may be compromised by biases from two main sources: 1. the assessment tool (SHARE-FI) is an effective method for screening frailty in primary care settings [42], but it tends to overestimate prevalence [43]; 2. the self-reported data are inevitably subject to recall bias.

## Conclusion

Our observational study found that physical activity frequency decreased with age in middle-aged and older adults. The incidence of frailty increases with decreased PA frequency in middle-aged and older adults. Additionally, women need to pay more attention to frailty prevention.

## Data Availability

The data that support the findings of this study are available from SHARE Wave 7 (10.6103/SHARE.w7.800) and Wave 7 (10.6103/SHARE.w9.900), but restrictions apply to the availability of these data, which were used under license for the current study, and so are not publicly available. Data are, however, available from the authors upon reasonable request and with permission of SHARE project.
